# The influence of self-cycling fermentation long- and short-cycle schemes on *Saccharomyces cerevisiae* and *Escherichia coli*

**DOI:** 10.1038/s41598-022-16831-x

**Published:** 2022-08-01

**Authors:** Yusheng Tan, Lisa Y. Stein, Dominic Sauvageau

**Affiliations:** 1 Department of Chemical and Materials Engineering, University of Alberta, Edmonton, AB Canada; 2 Department of Biological Sciences, University of Alberta, Edmonton, AB Canada

**Keywords:** Biotechnology, Microbiology, Molecular biology

## Abstract

Self-cycling fermentation (SCF), a cyclic process in which cells, on average, divide once per cycle, has been shown to lead to whole-culture synchronization and improvements in productivity during bioconversion. Previous studies have shown that the completion of synchronized cell replication sometimes occurs simultaneously with depletion of the limiting nutrient. However, cases in which the end of cell doubling occurred before limiting nutrient exhaustion were also observed. In order to better understand the impact of these patterns on bioprocessing, we investigated the growth of *Saccharomyces cerevisiae* and *Escherichia coli* in long- and short-cycle SCF strategies. Three characteristic events were identified during SCF cycles: (1) an optimum in control parameters, (2) the time of completion of synchronized cell division, and (3) the depletion or plateau of the limiting nutrient. Results from this study and literature led to the identification of three potential trends in SCF cycles: (A) co-occurrence of the three key events, (B) cell replication ending prior to the co-occurrence of the other two events, and (C) depletion or plateau of the limiting nutrient occurring later than the co-occurrence of the other two events. Based on these observations, microbial physiological differences were analyzed and a novel definition for SCF is proposed.

## Introduction

Self-cycling fermentation (SCF) is an advanced fermentation technique that improves productivity in many bioconversion processes^1–4^. It is a semi-continuous, unsteady-state, cyclical mode of operation, in which cycles are triggered upon depletion of a limiting nutrient^5,6^. Many metabolism- and growth-related parameters—including dissolved oxygen (DO), carbon dioxide evolution rate (CER), oxidation–reduction potential (ORP) and exit gas mass flow rate—have been used as control parameters for the automated feedback-control necessary for SCF cycling^7–9^. Cycling consists of the removal (harvest) of exactly one half of the working volume before replenishing with the same amount of fresh medium^5,6^.

The increased productivity demonstrated in many SCF studies^1,2,4,6,8,10,11^ is strongly related to the operational characteristics of this semi-continuous process. Compared to a conventional batch reactor (BR), SCF cycles have negligible lag or stationary phases. On the other hand, in contrast to chemostats, SCF may greatly minimize nutrient waste. Moreover, SCF operation has shown strong potential for degradation of pollutants when they were used as limiting carbon or nitrogen sources^12–16^.

SCF and continuous phasing^17–20^, its forebearer, share many similarities. One is the entrainment mechanism leading to the periodic availability of essential nutrients inducing synchronization^21^. This leads to sharp increases in cell count within a given cycle observed in both continuous phasing and SCF^21^. DNA content and cell size, determined through flow cytometry, have been used to validate synchrony during continuous phasing of bacteria^22^, while transcriptomic patterns has been used to confirm synchrony during SCF of yeast^23^. Many relevant trends were observed in the latter study: most genes related to DNA replication and half the genes associated with the yeast cell cycle were significantly up-regulated at the same point, early in SCF cycles^23^.

The incorporation of a feedback control loop to trigger cycling at the exhaustion of the limiting nutrient in SCF is a major improvement to continuous phasing^7^. In a large number of SCF studies, the cycle time was found to be equal to the doubling time of the microorganism growing under BR with the same nutrient conditions^6,10,13,15,18,24^. Subsequently, SCF cycle time was used to reflect the nutrient quality of the environment in a number of physiological studies; wherein a shorter cycle time suggested more efficient cell replication and thus more beneficial nutrient conditions^7^.

Although many SCF studies have depicted the co-occurrence of the completion of the cell cycle with the depletion of the limiting nutrient and a minimum in DO (or maximum in CER)^6,10,13,15,18,24^, recent studies conducted with *Saccharomyces cerevisiae* and *Escherichia coli* showed that the end of synchronized cell division (corresponding to a maximum in CER) could also occur before depletion of the limiting nutrient^2,3,5,23^. This suggests SCF can lead to more than one set of physiological patterns in cultures, and underscores the fact that the traditional description of SCF does not include these cases. This can greatly impact the physiology, operation, performance, and outputs of these cultures. To remediate this situation, we show that SCF long- and short-cycle strategies can both lead to stable cyclic operation of *S. cerevisiae* and *E. coli* with enhanced volumetric biomass productivity. We also investigated the transcriptional shifts of selected cyclin genes of *S. cerevisiae* growing in short cycles. These results, combined with previous studies, led to the identification of three possible trends—based on the occurrence of optima in the control parameter, end of cell division, and depletion or plateau of the limiting nutrient—during SCF operation. These results enable an enhanced understanding of the cellular processes during SCF, highlight the potential of SCF as a research tool to study cell physiology, and provide guidance for the development of more efficient bioconversion processes.

## Results

### *S. cerevisiae* grown in SCF long- and short-cycle schemes

**Figure 1:**
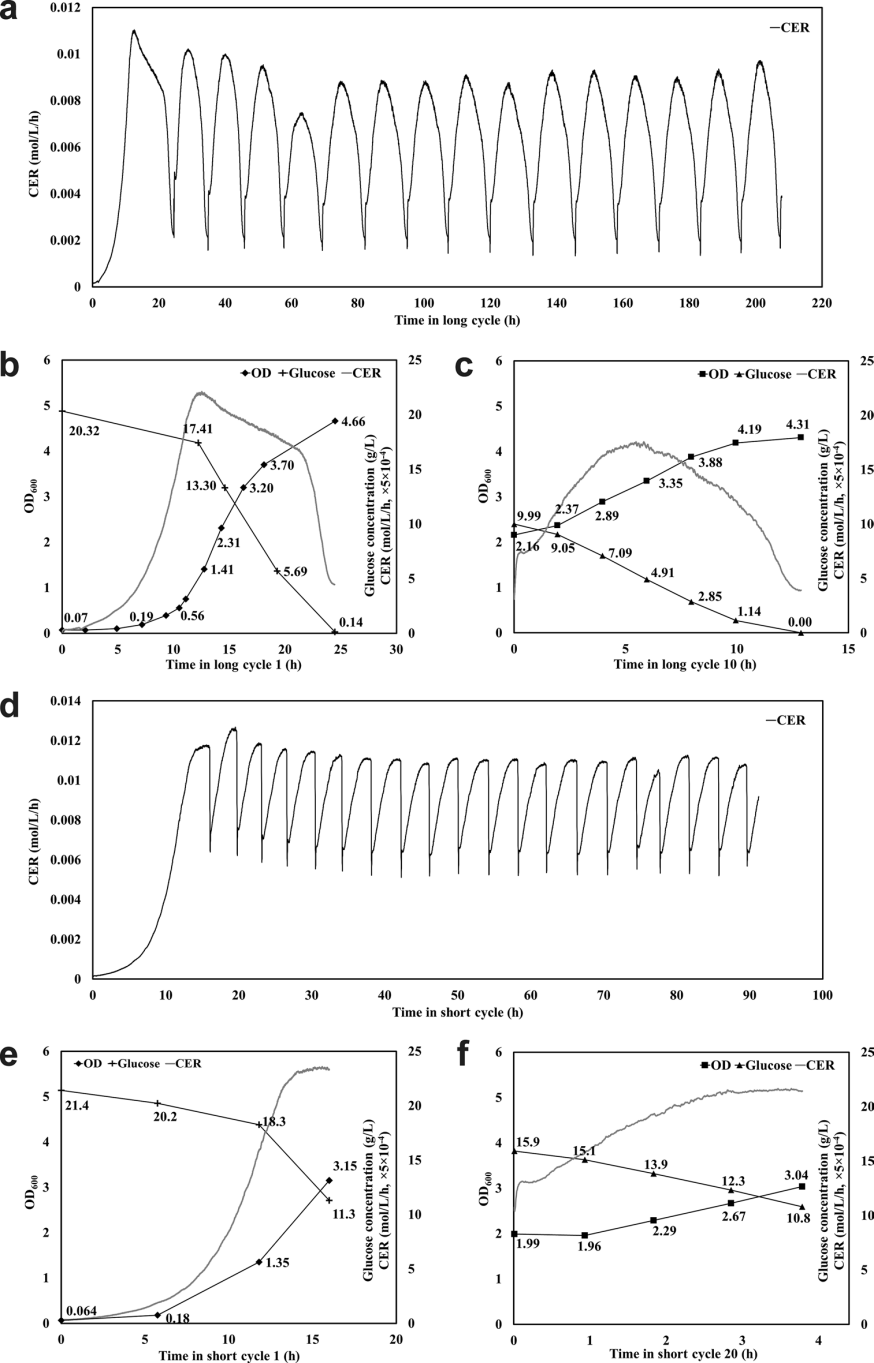
*S. cerevisiae* grown under SCF long- and short-cycle schemes. Long-cycle operation: (**a**) Carbon dioxide evolution rate (CER), (**b**) intracycle OD_600_, glucose concentration and CER in long cycle 1 (BR), (**c**) intracycle OD_600_, glucose concentration and CER in long cycle 10. Short-cycle operation: (**d**) CER, (**e**) intracycle OD_600,_ glucose concentration and CER in short cycle 1 (BR), (**f**) intracycle OD_600_, glucose concentration and CER in short cycle 20.

*S. cerevisiae* was cultivated under SCF long- and short-cycle schemes; the former cycled when the decreasing CER plateaued, whereas the short-cycle scheme was triggered upon reaching a maximum in CER (Fig. 1a,d; replicate results in Supplementary Fig. S1a,b). Based on their CER profiles, both long- and short-cycle modes of operation were highly stable and reproducible from cycle 2 onwards (Fig. 1a,d). However, there was a substantial difference in the shapes of CER curves (no decrease seen in short cycles), the CER maximum (9.2 ± 0.6 mmol/L/h in long cycles and 11.2 ± 0.5 mmol/L/h in short cycles) and the mean CER per cycle (integrated CER per cycle time; 6.7 ± 0.4 mmol/L/h for long cycles and 9.7 ± 0.4 mmol/L/h for short ones) (Fig. 1a,d and Supplementary Fig. S2). Cycle times were significantly different, with an average cycle time of 12.11 ± 0.73 h for long cycles and 3.80 ± 0.27 h for short cycles (Fig. 1a,d and Supplementary Fig. S2).

Figure 1b,c depict intracycle biomass accumulation (assessed by optical density (OD_600_)), glucose consumption and CER in cycles 1 and 10, respectively, of long-cycle operation. The CER maximum in cycle 1 occurred at the transition point between exponential phase and diauxic shift (Fig. 1b). Glucose, the limiting nutrient, was depleted at the end of both cycles, consistent with the occurrence of the condition for cycling (flattening of CER).

In the short-cycle operation, similar patterns in intracycle OD_600_ and glucose concentration were observed in cycles 1 and 20 (Fig. 1e,f). However, glucose was not exhausted by the end of the cycles (Fig. 1e,f) and, consequently, the end-of-cycle OD_600_ was lower than in the long-cycle counterparts. However, biomass yield was comparable between the long and short cycles − 0.22 and 0.21 L/g glucose, respectively (Fig. 1c,f; Table 1). On the other hand, volumetric productivity of *S. cerevisiae* cells was 0.17 h^−1^ in long cycle 10 and 0.28 h^−1^ in short cycle 20, a 1.6-fold increase (Fig. 1c,f; Table 1), and the glucose consumption rate was also found to be greater in short cycle 20 (0.78 g glucose/L/h and 1.31 g glucose/L/h for the long and short cycle, respectively; Fig. 1c,f).

**Table 1:** Biomass yield and volumetric productivity during SCF long- and short-cycle operation.

SCF operation	Yield of cells (L/g glucose)	Volumetric productivity of cells (h^−1^)
*S. cerevisiae* long-cycle operation (this study)	0.22	0.17
*S. cerevisiae* short-cycle operation (this study)	0.21	0.28
*E. coli* long-cycle operation (this study)	0.34	0.15
*E. coli* long-cycle operation (2010)^5^	0.23^a^	0.28^a^
*E. coli* short-cycle operation (this study)	0.63	0.42

^a^Values were calculated based on original figure found in^5^ using Eqs. (1) and (2).

### *S. cerevisiae* cell replication in SCF short-cycle scheme

**Figure 2:**
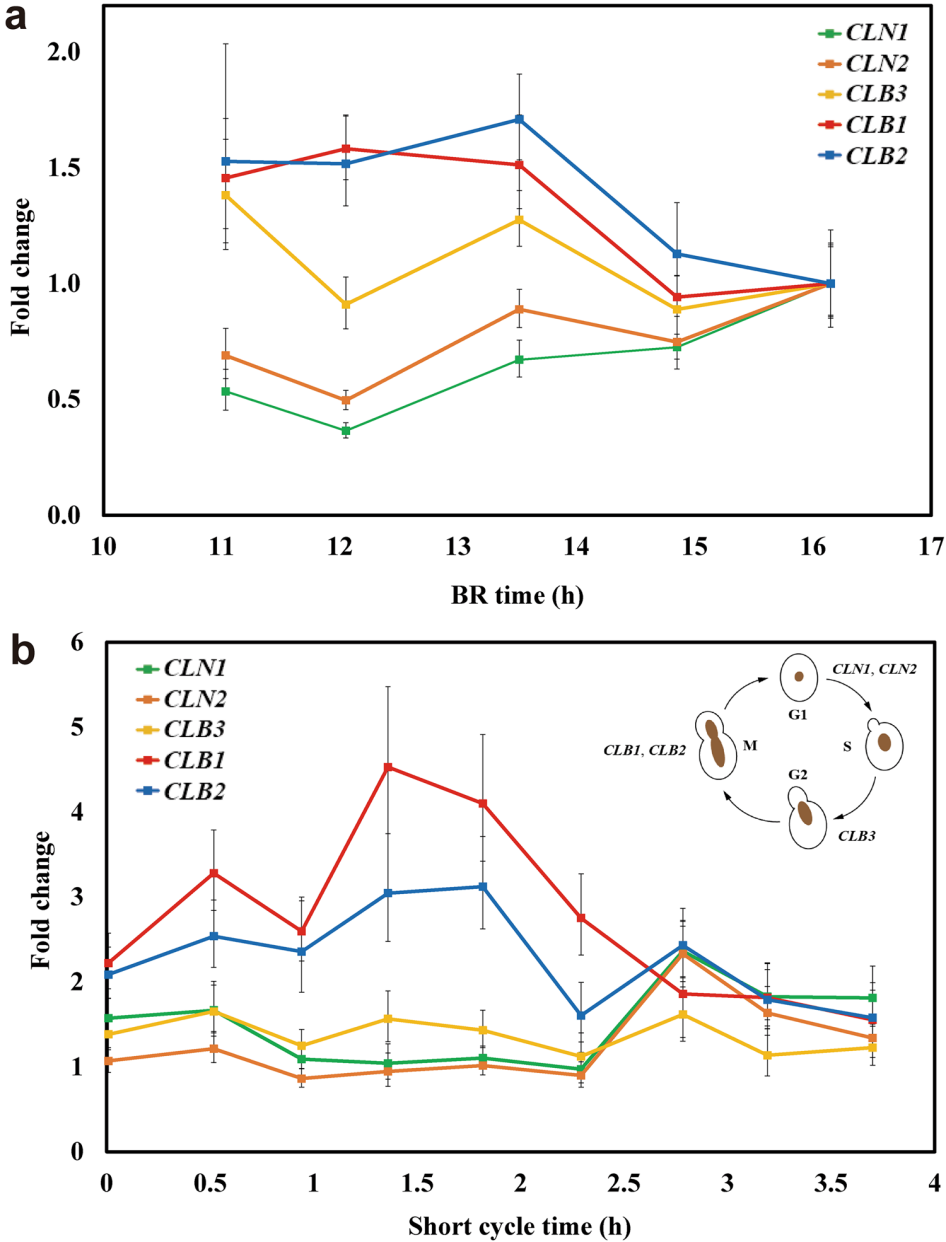
Relative expression of selected *S. cerevisiae* cyclin genes. (**a**) Fold changes of *CLN1*, *CLN2*, *CLB3*, *CLB1,* and *CLB2* during BR late-log phase. (**b**) Fold changes of the same cyclin genes during SCF short cycle 21 (the top-right schematic illustrates the expression sequence of the cyclin genes in regular cell cycle^25–28^). *ACT1* and *ALG9* were used as reference genes, and a sample collected at 16.2 h during BR was used as the reference sample. Error bars show one standard deviations (n = 3).

Relative expression (fold changes) of the cyclin genes, *CLN1*, *CLN2*, *CLB3*, *CLB1*, and *CLB2*, was determined for *S. cerevisiae* growing in SCF short cycles 1 and 21 using RT-qPCR (Fig. 2a,b; biological replicate results in Supplementary Fig. S3). Generally, fold changes of the cyclin genes during late-log phase in BR (short cycle 1) were not substantial: slight decreases in expression were found for *CLB1* and *CLB2*, and slight increases in expression were observed for *CLN1* and *CLN2* over the course of the BR (Fig. 2a). In contrast, during short cycle 21: (1) *CLB1* and *CLB2* (paralog genes) were significantly up-regulated in the early stages of the cycle until 2.8 h, with peaks in expression at approximately 1.4–1.8 h; (2) expression of *CLN1* and *CLN2* (paralog genes) was significantly up-regulated at 2.8 h; and (3) *CLB3* transcription remained relatively steady throughout the cycle (Fig. 2b).

Considering the sequential up-regulation of these cyclin genes, it can be established that some extent of synchrony was achieved over short-cycle operation. During a standard yeast cell cycle, *CLN1* and *CLN2* are up-regulated prior to *CLB1* and *CLB2*; *CLN1* and *CLN2* are expressed in G1 and S phases while *CLB1* and *CLB2* during M phase^25–28^. However, in short cycle 21, the up-regulation of *CLB1* and *CLB2* was shown to be earlier than that of *CLN1* and *CLN2*. This was indicative of partial (if not complete) synchronization of cell replication taking place from the middle of the short SCF cycles.

### *E. coli* grown in SCF long- and short-cycle schemes

**Figure 3:**
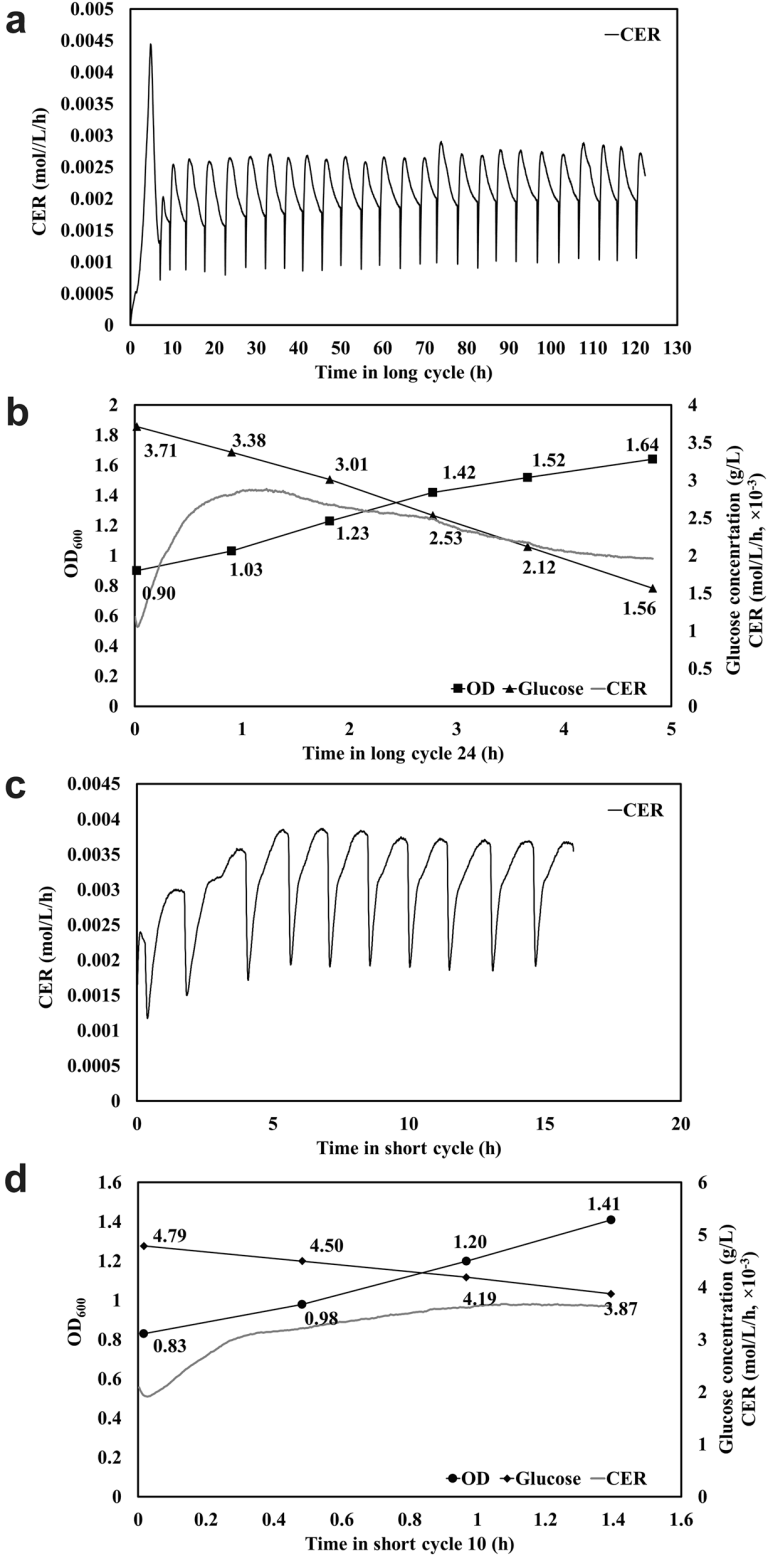
*E. coli* grown in SCF long-cycle and short-cycle schemes. Long-cycle scheme: (**a**) CER, and (**b**) intracycle OD_600_, glucose concentration and CER in long cycle 24. Short-cycle scheme: (**c**) CER, and (**d**) intracycle OD_600,_ glucose concentration and CER in short cycle 10.

*E. coli* MG1655 was grown under SCF long- and short-cycle operation, with cycling triggered once CER flattened and once CER reached a maximum, respectively. For SCF long cycles, a repeatable pattern of CER—consisting of a sharp increase followed by a decrease—was established directly after the second cycle (Fig. 3a; Supplementary Fig. S1c). The cycle time during long-cycle operation averaged 4.61 ± 0.32 h for cycles 3–26 (Fig. 3a and Supplementary Fig. S4). When the operation was tuned to the short cycle scheme, readaptation occurred within the first two cycles after the transition cycle, and a new stable pattern of CER was obtained (Fig. 3c; Supplementary Fig. S1d). The new CER pattern consisted of an increase to the maximum in CER which triggered cycling. The cycle time was reduced to 1.49 ± 0.07 h for short cycles 3–10 (Fig. 3c and Supplementary Fig. S4). Meanwhile, the CER maximum increased from 2.70 ± 0.09 to 3.77 ± 0.07 mmol/L/h and the mean CER per cycle significantly increased (from 2.20 ± 0.09 to 3.28 ± 0.04 mmol/L/h) once long cycles were switched to short cycles (Fig. 3a,c and Supplementary Fig. S4).

Regardless of the scheme used, the intracycle increase in OD_600_ and decrease in glucose concentration were linear (Fig. 3b,d). More biomass was accumulated in long cycle 24 (OD_600_ of 1.64 compared to 1.41 for short cycle 10), consistent with a greater amount of glucose consumed. As expected, the cycle time was also significantly greater in long-cycle operation (Fig. 3b,d). The *E. coli* yield was 0.34 L/g glucose in long cycle 24 compared to 0.63 L/g glucose for short cycle 10 (a 1.8-fold increase), and the biomass productivity for these cycles was 0.15 h^−1^ and 0.42 h^−1^ (a 2.7-fold increase), respectively (Fig. 3b,d; Table 1). The glucose consumption rate was also faster in short cycle 10 (0.66 g glucose/L/h compared to 0.44 g glucose/L/h in long cycle 24; Fig. 3b,d). It was also noted that, in the long-cycle scheme, glucose was not completely depleted at the end of the cycles (Fig. 3b). This is consistent with observations in an extended BR experiment (Supplementary Fig. S5), likely due to metabolic stalling or transitions between metabolic regimes.

## Discussion

### Improved productivity in SCF

In *S. cerevisiae* cultures undergoing SCF operation, long- and short-cycle operation led to similar yields but the latter led to a 1.6-fold increase in volumetric productivity (Table 1). On the other hand, in *E. coli* cultures, the short-cycle operation led to increases in both yield and productivity, by 1.8-fold and 2.7-fold, respectively (Table 1). In a previous study investigating *E. coli* ATCC 11303 growing in SCF long cycles^5^, the yield was found to be 0.23 L/g glucose, and the biomass productivity was 0.28 h^−1^ (Table 1). It was also noted that the average glucose consumption rate and mean CER per cycle were enhanced during short-cycle operation for both *S. cerevisiae* and *E. coli*, despite lower cell density (Figs. 1, 3 and Supplementary Figs. S2, S4)—indicating more glucose consumed and CO_2_ released per cell in short cycles. That is to say, cellular activity was generally more intense during SCF short cycles.

Significant improvements in productivity and metabolic activity highlight the advantages of the SCF short-cycle scheme over its long-cycle counterpart; however, both approaches have benefits over BR. In fact, increased productivity (compared to BR) has been shown in many long-cycle SCF experiments, including production of ethanol and shikimic acid using *S. cerevisiae*^3,4,8,23^ and production of bacteriophage and recombinant protein using *E. coli*^1,2^. It is likely that productivity could be further improved upon implementation of short-cycle SCF schemes.

### Cell replication during SCF long and short cycles

Multiple SCF studies have shown a link between the occurrence of an optimum in DO or CER and the completion of synchronized cell division^7^. In order for SCF operation to be stable, since it relies on the replacement of one half of the reactor content, the cell population must double (one generation) every cycle. If cells did not complete one round of cell replication per cycle, washout would occur, resulting in instability. Since the short-cycle operation was stable and repeatable in *S. cerevisiae* and *E. coli* cultures (Figs. 1d, 3c and Supplementary Figs. S1, S2, S4), we can infer that cell division was complete each cycle by the time CER reached a maximum. Step-wise cell count doubling, ending co-currently with a maximum in CER, was observed in previous SCF studies using long cycles with *S. cerevisiae* (the same strain as in the current study)^3,23^ and *E. coli* (ATCC 11303^5^ and CY15050^2^). This suggested that cell replication was indeed synchronized. Thirdly, prior work with *S. cerevisiae* showed significant up-regulation of genes related to DNA replication and of selected cyclin genes (*CLN1*, *CLN2*, *CLB3*, *CLB1*, and *CLB2*) in the first half of long SCF cycles^23^. This suggests little to no replication activity took place after the maximum in CER was reached.

The expression profiles of cyclin genes in *S. cerevisiae* undergoing short SCF cycle (Fig. 2) provide evidence of at least some level of cell synchrony. It is interesting to note that, compared to expression of the same genes in the same strain undergoing long SCF cycles^23^, the amplitude of the differential expression observed was smaller during short cycles (Fig. 2b). This was likely due to the incomplete utilization of glucose, resulting in a tamer entrainment effect (an effect leading to the periodic availability of essential nutrients inducing synchronization^21^) during short cycles. Secondly, the sequence of cyclin genes expression suggested that the cell replication of partially synchronized populations started from the middle of the short cycles and was completed at the same point in the subsequent cycle (with cell division occurring over a shorter period). *CLN1* and *CLN2* were expressed later than *CLB1* and *CLB2* during SCF short cycles (Fig. 2b)—an inverse sequence compared to the standard yeast cell cycle^27,28^ and to the sequence observed in long SCF cycles^23^. This unexpected, distinct, cycle-spanning cell replication pattern in short-cycle SCF operation could have been caused by forces other than the oscillation of glucose concentration, as the nutrient cycle itself is expected to lead to an alignment between the start of SCF cycles and that of the cell cycle^23^.

It appears that yeast cell replication did not present the same intra-cycle pattern during long SCF cycles. For one, as mentioned above, there was no substantial expression of the selected cyclin genes during the second half of the long cycles^23^, suggesting little to no replication activity over that period. Moreover, the onsets of long SCF cycles and of the yeast cell cycle were aligned, as suggested by the sequential expression of the cyclin genes during the first half of long cycles (consistent with the standard yeast cell cycle; *CLN1* and *CLN2* expressed earlier than *CLB1* and *CLB2*)^23^. Furthermore, the cycle time of long cycles was more than twice the doubling time of *S. cerevisiae* in the same nutrient conditions.

Continuous operation with limited carbon feeding after starvation resulted in robust oscillations in the profiles of DO, transcriptomics, and metabolomics, suggesting mechanisms encompassing the cell and metabolic cycles^29–31^. While SCF operation does not involve significant nutrient limitation, previous transcriptomic work also demonstrated significant changes in regulatory patterns during each SCF cycle^23^. For instance, towards the end of *S. cerevisiae* long cycles, the citrate cycle, oxidative phosphorylation, and gluconeogenesis pathways were highly up-regulated. Future work will be allocated on measuring more metabolic patterns during SCF cycles and comparing SCF operation with carbon-limited continuous operation.

### Three trends in SCF

**Figure 4:**
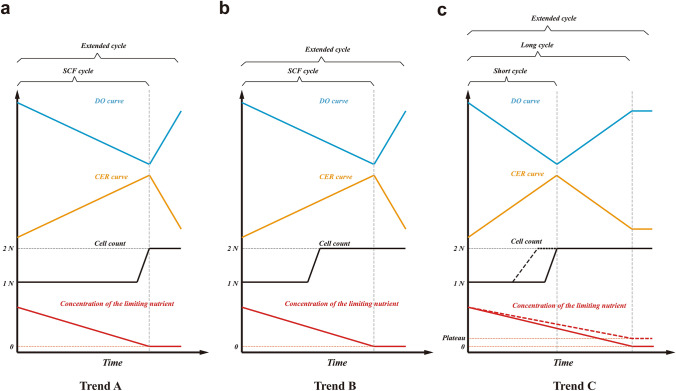
Schematic of the conceptual trends in characteristic events during SCF. DO curve (for aerobic conditions) is shown in blue, CER curve (for aerobic or anaerobic conditions) in orange, cell count in black, and concentration of the limiting nutrient in red. Straight lines are used to describe general trends. (**a**) Trend A: Optimum in control parameter (DO minimum or CER maximum), the end of synchronized division, and the depletion of the limiting nutrient co-occur at the end of the SCF cycle. An extended cycle allows for a delay in cycling. (**b**) Trend B: Optimum in control parameter (DO minimum or CER maximum) and the depletion of the limiting nutrient co-occur at the end of the SCF cycle, but synchronized cell division ends in the middle of the cycle. An extended cycle allows for a delay in cycling. (**c**) Trend C: DO or CER plateaus as limiting nutrient becomes depleted (or reaches its own plateau, the red dashed line) at the end of the SCF long cycle, but synchronized cell division ends in the middle of the long cycle (corresponding to an optimum in DO or CER). In trend C, an SCF short cycle can be implemented when cycling is done at the time of the optimum in control parameters but partially synchronized cell replication (the black dashed line) starts and ends in the middle of the short cycle. The limiting nutrient is not depleted by the end of the short cycle. An extended cycle allows for a delay in cycling beyond the end of a long cycle.

When surveying SCF studies, three different trends, based on significant differences in the occurrence of key events, take shape. These key events are (1) the time of the optimum in the control parameter (e.g., DO minimum, CER maximum or ORP inflection point), (2) the time of completion of synchronized cell division, and (3) the depletion or plateau of the limiting nutrient. The three trends based on these events are summarized in Fig. 4.

Nitrogen or carbon sources are frequently set as the limiting nutrients dictating the cycling of SCF operation. Control parameters used to establish cycling conditions have included DO, CER, and ORP^7^. Mass flow rate of the exit gas has also been used for SCF of *S. cerevisiae* Superstart™ producing ethanol^8^ and was a direct reflection of CER under anaerobic conditions. In studies of *Pseudomonas putida* ATCC 12633 degrading phenol^12^ and *Acinetobacter calcoaceticus* RAG-1 ATCC 31012 grown on hexadecane^32^, CER patterns were found to mirror DO patterns, and the CER maximum aligned with the DO minimum. Under aerobic conditions, the relationship between CER and DO generally holds, with exceptions. However, ORP patterns during SCF operation are generally more complex than other parameters. For example, ORP increased for *P. putida* ATCC 12633 degrading toluene^16^, but decreased when *Pseudomonas denitrificans* ATCC 13867 was used for the removal of oxidized nitrogen^15^. The presence and absence of oxygen in these two studies were likely responsible for these diverging patterns. Overall, during SCF operation, the minimum in DO coincides with the maximum in CER (under aerobic conditions), and the ORP inflection point occurs near this point^15,16^.

Many early SCF studies exhibited Trend A (Fig. 4a), in which the depletion of the limiting nutrient coincided with the characteristic point in the control parameter (OD, CER or ORP) and with the completion of synchronized cell division. SCF cycling was triggered upon this concurrence unless an extended cycle strategy was applied. The first implementation of SCF, for *B. subtilis* ATCC 21332, showed the minimum in DO corresponded with the depletion of the nitrogen source and the end of OD doubling^18^. The end of synchronized cell doubling (a step-wise increase in cell count) co-occurred with nitrogen source exhaustion and with a minimum in DO when SCF was used to produce sophorolipids by *Candida bombicola* ATCC 22214^24^ and citric acid by *Candida lipolytica* ATCC 20390^10^. The same pattern was observed for *A. calcoaceticus* RAG-1 ATCC 31012 grown on ethanol^6^ and for *P. putida* ATCC 12633 degrading aromatic compounds^13^. Similarly, when *P. denitrificans* ATCC 13867 was used to degrade oxidized nitrogen sources in SCF, the end of cell dry weight doubling corresponded to the inflection point in ORP and to nitrogen depletion^15^.

To that point, the reliability of Trend A (Fig. 4a) had been considered universal. For example, in studies tackling hydrocarbon degradation using *A. calcoaceticus* RAG-1 ATCC 31012 and cultivating *B. subtilis* ATCC 21332, the authors directly took the equivalence of SCF cycle time and cell doubling time as a default^14,17^. However, this was only true when synchronized cell division was terminated upon the initiation of SCF cycling^6,10,13,24^. It should also be noted that the end point of the doubling of OD or dry weight does not necessarily represent the end of cell doubling. These can be decoupled and display different trends, especially in synchronized populations for which the cell count increases in a step-wise manner, while OD and dry weight display continuous, near-linear increases^2,33^.

However, when growing *Alcaligenes eutrophus* DSM 545 producing polyhydroxybutyrate (PHB) and growing *B. subtilis* ATCC 10774 under SCF operation, synchronized cell division was completed much earlier—in the middle of the SCF cycles—than the concomitant minimum in DO and depletion of nitrogen sources^33,34^. These SCF cultures are representative of Trend B (Fig. 4b).

Compared to Trends A and B, the scenario observed in a study investigating biosurfactant production using *Corynebacterium alkanolyticum* ATCC 21511 growing on hexadecane in SCF was substantially different^35^. The minimum in DO and the completion of synchronized cell division occurred concomitantly, but a considerable amount of carbon source remained. Similarly, recent SCF work using engineered *S. cerevisiae* CEN.PK 113-1A Matα, *E. coli* ATCC 11303 and *E. coli* CY15050 depicted an identical trend—cell count doubled step-wise at the maximum in CER (at the cycle midpoint), but glucose, the limiting nutrient, was only exhausted once the decrease in CER flattened (at the end of the cycles)^2,3,5,23^. In ethanol production using *S. cerevisiae* Superstart™ undergoing anaerobic SCF, glucose was depleted upon the time the exit gas mass flow rate (a proxy for CER) decreased and stabilized, though cell counts were not reported due to clumping of the yeast cells^8^. As mentioned earlier, the same trend was also observed in the present study when cultivating engineered *S. cerevisiae* CEN.PK 113-1A Matα or *E. coli* MG1655 (with glucose reaching a plateau when the latter underwent long-cycle SCF). Transcriptional evidence during *S. cerevisiae* SCF short cycles (Fig. 2b) revealed a likely cell replication pattern under this operation scheme: a partially synchronized cell cycle starting and ending in the middle of short cycles. All these studies are representative of Trend C (Fig. 4c).

The discrepancies amongst the three major trends were likely derived from intrinsic differences in the microorganisms and nutrient environments used. *A. eutrophus* and *B. subtilis* ATCC 10774 (following Trend B), and *C. alkanolyticum*, *E. coli* and *S. cerevisiae* (following Trend C) likely sensed nutrient conditions more actively and adopted a feed-forward strategy—in which cells proactively sensed external changes and regulated gene transcription and expression prior to the alteration of the growth rate^36^. From a growth strategy perspective, it seems these synchronized cultures completed one cell cycle but did not continue the proliferation at the expense of the remaining limiting nutrient (Fig. 4b,c). On the contrary, for a number of microorganisms following Trend A, all the available limiting nutrient was used in completing cell doubling (Fig. 4a). The difference between *A. eutrophus* and *B. subtilis* ATCC 10774 in Trend B, and *C. alkanolyticum*, *E. coli* and *S. cerevisiae* in Trend C is expected to lie in the respiratory intensity between the end of the cell cycle and the time at which the limiting nutrient was depleted or reached a plateau. For the Trend B microbes, the intensity of respiration increased even after synchronized cell replication. Therefore, the optimum in the control parameter (DO minimum) co-occurred with the exhaustion of the limiting nutrient but not with the end of cell doubling (Fig. 4b). For microbes displaying Trend C, respiration slowed significantly after synchronized cell replication (during the consumption of the residual limiting nutrient), and therefore CER maximum or DO minimum occurred at the completion of synchronized cell doubling but not at the depletion or plateau of the limiting nutrient (Fig. 4c).

Physiological differences in strategies for nutrient use, proliferation, and respiratory intensity are hence revealed during SCF operation, suggesting that this method could be helpful in studying cell physiology. It is also noted that synchrony helps with these explorations: trends reflected by synchronous populations would be more reflective of intrinsic physiological properties.

Different nutrient conditions may lead to different physiological responses and affect SCF trends. For example, the use of different limiting nutrients—nitrogen- or carbon-sources—in a continuous phased culture tremendously affected the time of completion of synchronized cell replication of *Candida utilis* Y-900 when the cycle time was set to 4, 6, 8, and 12 h^37^. Further studies on this topic could lead to more in-depth understanding of the physiological patterns during SCF.

### A novel definition of SCF

Limiting nutrient depletion has been one of the original premises of SCF, but a broader picture is emerging. Trend C, observed in a growing number of studies, suggests a deviation from the original description of SCF–it does not necessarily require limiting nutrient depletion. Consequently, a novel definition of SCF is proposed below, taking into consideration all the scenarios presented in Fig. 4. This new SCF definition excludes the requirements of limiting nutrient depletion and joint occurrence of all three key events.

SCF is a semi-continuous fermentation approach that allows the completion of one generation of microbial cell replication during each cycle. The cycling procedure comprises harvesting precisely one half of the working volume and then replenishing with the equivalent amount of fresh medium. Automated cycling is dictated by microbial growth and metabolic activity and is triggered based on monitoring one or more growth- and/or metabolism-associated parameters (e.g., DO, CER, ORP, exit gas mass flow rate, etc.). SCF cycling takes place directly after the completion of one generation of cell proliferation or with a delay, depending on the microorganism, the initial nutrient conditions, and the conditions for cycling being implemented. SCF cycling is not necessarily related to the time at which the limiting nutrient is depleted or reaches a plateau. If limiting nutrient depletion or a plateau does not co-occur with the cell cycle completion, we identify SCF operation that cycles in advance of exhaustion or a plateau of the limiting nutrient as “short cycle”; and correspondingly, SCF operation that cycles upon depletion or a plateau of the limiting nutrient as “long cycle” (Fig. 4). “Extended cycle” is generally referred to as SCF operation that cycles beyond exhaustion or a plateau of the limiting nutrient.

## Conclusions

Previous SCF operation of *S. cerevisiae* and *E. coli* triggered cycling upon glucose depletion when the CER flattened, obtaining greater productivity compared to BR. In the present study, cultures of *S. cerevisiae* and *E. coli* were cycled once CER reached a maximum, which led to stable and reproducible short cycles (short-cycle scheme). This led to a notable improvement in volumetric biomass productivity compared to the long-cycle scheme. Transcriptional analysis of selected *S. cerevisiae* cyclin genes during SCF short cycles inferred a cycle-spanning mode of cell replication.

A thorough review of previous SCF highlighted three typical trends in the occurrence of three SCF characteristic events, (1) the optimum in control parameters (e.g., CER maximum), (2) the completion of synchronized cell division, and (3) the depletion or plateau of the limiting nutrient. A novel description of SCF was hence proposed to include all scenarios of SCF operation and clear definitions for SCF “short cycle”, “long cycle”, and “extended cycle”.

This work highlights the potential of SCF as a research tool to explore microbial physiological properties—including nutrient use, proliferation strategies, and respiration intensity. It also demonstrates the potential in using short-cycle schemes to further improve the performance of bioconversion. Finally, it consolidates and deepens our understanding of the SCF technique and its influences on microbial populations, providing a solid framework to guide further design and implementation of SCF-based processes.

## Methods and materials

### Strains, media, and pre-cultures

*Escherichia coli* MG1655 (CGSC 6300) was used in *E. coli* experiments. Luria–Bertani (LB) broth (all chemicals used in this study were purchased from Fisher Scientific and Sigma Aldrich, Canada) was used for agar plates (1.5% w/v of agar). Semi-defined liquid medium, containing 6 g/L sodium phosphate dibasic, 4 g/L ammonium nitrate, 4 g/L potassium phosphate monobasic, 0.014 g/L disodium EDTA, 0.05 g/L yeast extract, 0.01 g/L calcium chloride dihydrate, 0.01 g/L iron sulfate heptahydrate, 6 g/L glucose, and 0.2 g/L magnesium sulfate heptahydrate, was used in Erlenmeyer flasks and fermenters. Pre-cultures were grown in 250-mL Erlenmeyer flasks at 37 °C, 250 rpm for 12 h. Approximately 4 × 10^10^ cells (10 mL) were withdrawn from pre-cultures and used to inoculate the 1-L fermenter working volume to achieve 1% v/v inoculation.

An engineered *Saccharomyces cerevisiae*^38^, genetically modified to overproduce shikimic acid based on parental strain CEN.PK 113-1A *MAT*α, was kindly provided by Prof. Vincent Martin at Concordia University. *E. coli AROB*, *E. coli AROD*, and the feedback-resistant variant of *S. cerevisiae ARO4* (*ARO4* K229L) were introduced using a pYES plasmid with *URA3* for auxotrophic selection^38^. 1.92 g/L yeast synthetic drop-out medium excluding uracil was used for auxotrophic selection on agar plates (1.5% w/v of agar). 6.7 g/L yeast nitrogen base (YNB) without amino acids and 20 g/L dextrose comprised liquid medium. Pre-cultures were grown in Erlenmeyer flasks at 30 °C and 150 rpm for 48 h. Approximately 8 × 10^8^ cells (10 mL) from pre-cultures were added to the 1-L working volume in the fermenter to achieve 1% v/v inoculation.

### SCF configuration and operation

The SCF configuration was previously described in^5,23^; herein a 2-L stainless steel fermenter (10.5 cm I.D.) was used. The feed system included a 10-L carboy (Nalgene, Fisher Scientific) containing fresh medium, a peristaltic pump (77201-60, Cole Parmer), a solenoid valve (SV125, Omega), and a glass isolator. The harvesting system consisted of a solenoid valve (SV125, Omega) and a 10-L harvest carboy (Nalgene, Fisher Scientific). Air was supplied by passing through an air regulator (R07-200-RGKA, Norgren), a sterilized water bottle (for stabilization and humidification), a rotameter (03294-20, Cole Parmer), and a HEPA filter (Whatman). Exit gas flew through a glass condenser and a HEPA filter (Whatman). Carbon dioxide (CO_2_) in the exit gas was measured with an in-line CO_2_ gas sensor (CO_2_-BTA, Vernier) located after the filter. Precise volume control during cycling was realized using high-level and low-level optical sensors (ELS-900 series, Gems Sensors) at 1 L and 0.5 L, respectively. The temperature was monitored and controlled using a K-type thermocouple (GKQSS-18G-10, Omega) and a cartridge heater (CIR-1032/120 V, Omega). Real-time data of cycle time, temperature, carbon dioxide evolution rate (CER, based on CO_2_ concentration in the exit gas), and the first derivatives of CER over 20 min and 60 min (referred to as short dCER and long dCER) were monitored and recorded by LabView (National Instruments) via an OPTO 22 data acquisition board. A LabView program was used to control conditions and automate cycling.

The fermenter temperature was maintained at 37 °C during bacterial growth and 30 °C during yeast growth. Agitation at 250 rpm with a Rushton impeller (4-cm diameter) and aeration at 400 mL/min for *E. coli* and 845 mL/min for *S. cerevisiae* provided sufficient mixing and aerobic conditions. During the SCF cycling procedure, agitation was ceased to maintain liquid level stability. Cell culture drainage driven by gravity stopped when the liquid level reached the low-level sensor. Fresh medium was then pumped into the bioreactor until the 1-L working volume was reached.

The following conditions were used to trigger automated cycling. For the *E. coli* SCF long cycle operation: (1) cycle time was greater than 90 min; (2) the absolute value of short dCER was less than 0.02 ppm/min; (3) long dCER was less than 0. For the *E. coli* SCF short cycle scheme: (1) cycle time was greater than 60 min; (2) short dCER was less than -0.02 ppm/min. For the *S. cerevisiae* SCF long cycle scheme: (1) cycle time was greater than 300 min; (2) CER was less than 3000 ppm; (3) the absolute value of short dCER was less than 0.05 ppm/min; (4) long dCER was less than 0. For the *S. cerevisiae* SCF short cycle strategy: (1) cycle time was greater than 110 min; (2) short dCER was less than -0.02 ppm/min; (3) CER was more than 3000 ppm.

### Batch reactor configuration and operation

Supplementary Fig. S6 depicts the BR set-up, which was adapted from the SCF set-up and used when BR operation was decoupled from SCF operation in cultivating *E. coli* (results shown in Supplementary Fig. S5). Cultivation conditions during BR were congruent with those used for SCF operation. Additionally, the first cycles of SCF operation are analogues of BR.

### Measurement of optical density, glucose, ammonium, nitrate and nitrite

A spectrophotometer (Ultrospec 50, Biochrom) was used to measure optical density of culture samples at a wavelength of 600 nm (OD_600_).

Glucose concentration was determined using the reducing sugar method^39^. Dinitrosalicylic acid (DNS) reagent was prepared, containing 10 g dinitrosalicyclic acid, 2 g phenol, 0.5 g sodium sulfite, and 10 g sodium hydroxide in 1 L deionized water. 20 µL of the filtered samples were mixed with 140 µL of DNS reagent, followed by a 5-min incubation at 95 °C. Samples were then cooled on ice for 5 min to stop the reactions. After that, 840 µL of deionized water was added. Samples were finally measured through a spectrophotometer (Ultrospec 50, Biochrom) set to a wavelength of 540 nm. A standard curve, based on standards, was used for quantification.

Nitrogen measurements followed methods detailed in^40^. To measure ammonium, a solution of 12 g/L of sodium hydroxide was mixed with another containing 85 g/L sodium salicylate and 0.6 g/L sodium nitroprusside at a 2:1 volume ratio. 375 µL of this freshly prepared mixture was added to 750 µL of every sample. 150 µL of 0.2 g/L sodium dichloroisocyanurate was then added, followed by 30 min of incubation in a dark environment. After incubation, absorbance of samples was measured at 660 nm using a spectrophotometer (Ultrospec 50, Biochrom). A standard curve was established based on standard solutions and used for quantification. To measure nitrate and nitrite, 75 µL of a catalyst solution containing 35.4 mg/L copper sulfate pentahydrate and 0.9 g/L zinc sulfate monohydrate was added to 500 µL of every sample. Then, 75 µL of 40 g/L sodium hydroxide and 75 µL of 1.71 g/L hydrazine sulfate were added sequentially, and samples were incubated in the dark for 15 min. After incubation, 250 µL of 10 g/L sulfanilamide dissolved in 3.5 M hydrochloric acid and 75 µL of 1 g/L naphthylethylene diamine dichloride were added sequentially, and samples were incubated in the dark for an additional 10 min. Samples were finally assessed by measuring absorbance at 540 nm using a spectrophotometer (Ultrospec 50, Biochrom). A standard curve was established based on standard solutions and used for quantification.

### Calculation of yield and productivity for biomass production

Equations (1) and (2) were used to calculate the yield and productivity in the production of *S. cerevisiae* or *E. coli* cells.

1 $${Y}_{X/S}=\frac{\Delta {OD}_{600}}{-\Delta {c}_{S}}$$

2 $${r}_{P}=\frac{\Delta {OD}_{600}}{\Delta t}$$

${Y}_{X/S}$ is the yield of *S. cerevisiae* or *E. coli* biomass (assessed by OD_600_) on glucose in L/g glucose. ${r}_{P}$ represents the volumetric productivity of *S. cerevisiae* or *E. coli* biomass (assessed by OD_600_) in h^−1^. ${c}_{S}$ is the substrate concentration in g glucose/L. $t$ represents operation time in h.

### RT-qPCR experiments for *S. cerevisiae*

Samples (0.5 mL) were collected at multiple sampling points during *S. cerevisiae* BR and SCF operation. Cells were centrifuged (13,000 g, 2 min), and the supernatant was discarded. Total RNA purification was performed using a Masterpure Complete DNA and RNA Purification Kit (Lucigen). The main steps consisted of cell lysis, protein precipitation, nucleic acid recovery, and genomic DNA removal. The manufacturer’s instructions were followed with the following modifications: dithiothreitol (DTT) was added to 1 mM before cell lysis, and disodium EDTA (pH 8.5) was added to 2.5 mM at the end of the DNA removal step to cease the digestion by DNase I. After RNA extraction, a NanoDrop 1000 (Thermo Fisher) and a Bioanalyzer 2100 (Agilent) were used to measure the concentration, quality, and integrity of the total RNA samples. A High-Capacity cDNA Reverse Transcription Kit with RNase Inhibitor (Thermo Fisher) was used for reverse transcription, implementing random primers and a standard temperature program. qPCR experiments were carried out using PowerUp SYBR Green Master Mix (Thermo Fisher) in a QuantStudio 3 real-time PCR instrument (Thermo Fisher). Each condition was tested in triplicate. A BR sample collected at the transition point from late-log phase to diauxic shift (at 16.2 h) was utilized as the reference sample for all alignments. *ACT1* and *ALG9* were used as reference genes based on literature^41–43^. The following genes were selected to assess the yeast cell cycle^25–28^: *CLN1* and *CLN2* (up-regulated from G1 phase to early S phase), *CLB3* (expressed in late S phase and G2 phase), *CLB1* and *CLB2* (accumulating transcripts in mitotic phase). Primers were designed using Primer3^44^. Their sequences, amplicon sizes, and efficiencies determined via standard curve experiments are shown in Table 2. Relative gene expression levels were calculated using the double delta C_t_ method^45^.

**Table 2:** Primer sequences, amplicon sizes, and efficiencies for qPCR experiments.

Gene	Forward and reverse primers	Amplicon size (bp)	Efficiency (%)
*ACT1*	5ʹ-CTCGTGCTGTCTTCCCATCT-3ʹ	69	101.15
	5ʹ-TTTGACCCATACCGACCAT-3ʹ		
*ALG9*	5ʹ-ACATCGTCGCCCCAATAAA-3ʹ	132	92.69
	5ʹ-CGTAAAATGCTCTACCCAAAATCTT-3ʹ		
*CLN1*	5ʹ-CTCGTATTCCACGCCTTTCT-3ʹ	114	93.52
	5ʹ-CGTCCCAGTTCAGAGTATCCA-3ʹ		
*CLN2*	5ʹ-TTCCTCATCTCAAAGCCACA-3ʹ	130	93.93
	5ʹ-TGACTGCTGCTGACCAAATT-3ʹ		
*CLB1*	5ʹ-CTCAGCGGCAATGTTCCT-3ʹ	90	102.68
	5ʹ-GCCTTTGTGTAACCACCACT-3ʹ		
*CLB2*	5ʹ-TGCCTTTTCATTGCCTCTAA-3ʹ	77	89.35
	5ʹ-GCACCGTCTGTCTCTGATG-3ʹ		
*CLB3*	5ʹ-AGGATGAAGAAGAAGACCAGGA-3ʹ	69	105.86
	5ʹ-GCTCCCAGACCAATGTATCA-3ʹ		

## Supplementary Information

Supplementary Figures.

## Data Availability

All data generated or analyzed during this study are included in this published article (and its Supplementary Information files).

## References

[1] Sauvageau D, Cooper DG (2010). Two-stage, self-cycling process for the production of bacteriophages. Microb. Cell Fact..

[2] Storms ZJ, Brown T, Sauvageau D, Cooper DG (2012). Self-cycling operation increases productivity of recombinant protein in *Escherichia coli*. Biotechnol. Bioeng..

[3] Agustin RVC (2015). The impact of self-cycling fermentation on the production of shikimic acid in populations of engineered *Saccharomyces cerevisiae*.

[4] Wang J, Chae M, Sauvageau D, Bressler DC (2017). Improving ethanol productivity through self-cycling fermentation of yeast: a proof of concept. Biotechnol. Biofuels.

[5] Sauvageau D, Storms Z, Cooper DG (2010). Synchronized populations of *Escherichia coli* using simplified self-cycling fermentation. J. Biotechnol..

[6] Brown WA, Cooper DG (1991). Self-cycling fermentation applied to *Acinetobacter calcoaceticus* RAG-1. Appl. Environ. Microbiol..

[7] Brown WA (2001). The self-cycling fermentor—development, applications, and future opportunities. Recent Res. Dev. Biotechnol. Bioeng..

[8] Wang J, Chae M, Bressler DC, Sauvageau D (2020). Improved bioethanol productivity through gas flow rate-driven self-cycling fermentation. Biotechnol. Biofuels.

[9] Wang J, Chae M, Beyene D, Sauvageau D, Bressler DC (2021). Co-production of ethanol and cellulose nanocrystals through self-cycling fermentation of wood pulp hydrolysate. Bioresour. Technol..

[10] Wentworth SD, Cooper DG (1996). Self-cycling fermentation of a citric acid producing strain of *Candida lipolytica*. J. Ferment. Bioeng..

[11] Zenaitis MG, Cooper DG (1994). Antibiotic production by *Streptomyces aureofaciens* using self-cycling fermentation. Biotechnol. Bioeng..

[12] Hughes SM, Cooper DG (1996). Biodegradation of phenol using the self-cycling fermentation (SCF) process. Biotechnol. Bioeng..

[13] Sarkis BE, Cooper DG (1994). Biodegradation of aromatic compounds in a self-cycling fermenter (SCF). Can. J. Chem. Eng..

[14] Brown WA, Cooper DG (1992). Hydrocarbon degradation by *Acinetobacter calcoaceticus* RAG-1 using the self-cycling fermentation technique. Biotechnol. Bioeng..

[15] Brown WA, Cooper DG, Liss SN (1999). Adapting the self-cycling fermentor to anoxic conditions. Environ. Sci. Technol..

[16] Brown WA, Cooper DG, Liss SN (2000). Toluene removal in an automated cyclical bioreactor. Biotechnol. Prog..

[17] Sheppard JD, Cooper DG (1991). The response of *Bacillus subtilis* ATCC 21332 to manganese during continuous-phased growth. Appl. Microbiol. Biotechnol..

[18] Sheppard JD, Cooper DG (1990). Development of computerized feedback control for the continuous phasing of *Bacillus subtilis*. Biotechnol. Bioeng..

[19] Dawson PSS (1965). Continuous phased growth, with a modified chemostat. Can. J. Microbiol..

[20] Dawson PSS (1972). Continuously synchronised growth. J. Appl. Chem. Biotechnol..

[21] Sheppard JD, Dawson PSS (1999). Cell synchrony and periodic behaviour in yeast populations. Can. J. Chem. Eng..

[22] Fritsch M, Starruß J, Loesche A, Mueller S, Bley T (2005). Cell cycle synchronization of *Cupriavidus necator* by continuous phasing measured via flow cytometry. Biotechnol. Bioeng..

[23] Tan Y, Agustin RVC, Stein LY, Sauvageau D (2021). Transcriptomic analysis of synchrony and productivity in self-cycling fermentation of engineered yeast producing shikimic acid. Biotechnol. Rep..

[24] McCaffrey WC, Cooper DG (1995). Sophorolipids production by *Candida bombicola* using self-cycling fermentation. J. Ferment. Bioeng..

[25] Feldmann, H. *Yeast: Molecular and Cell Biology*. *Wiley-Blackwell* (Wiley-Blackwell, 2012).

[26] Futcher B (1996). Cyclins and the wiring of the yeast cell cycle. Yeast.

[27] Cho RJ (1998). A genome-wide transcriptional analysis of the mitotic cell cycle. Mol. Cell.

[28] Fitch I (1992). Characterization of four B-type cyclin genes of the budding yeast *Saccharomyces cerevisiae*. Mol. Biol. Cell.

[29] Tu, B. P., Kudlicki, A., Rowicka, M. & McKnight, S. L. Logic of the yeast metabolic cycle: Temporal compartmentalization of cellular processes. *Science (80-. ).* **310**, 1152–1158 (2005). 10.1126/science.1120499 16254148

[30] Tu BP (2007). Cyclic changes in metabolic state during the life of a yeast cell. Proc. Natl. Acad. Sci. U. S. A..

[31] Murray DB, Beckmann M, Kitano H (2007). Regulation of yeast oscillatory dynamics. Proc. Natl. Acad. Sci. U. S. A..

[32] van Walsum GP, Cooper DG (1993). Self-cycling fermentation in a stirred tank reactor. Biotechnol. Bioeng..

[33] Marchessault P, Sheppard JD (1997). Application of self-cycling fermentation technique to the production of poly-β-hydroxybutyrate. Biotechnol. Bioeng..

[34] Sheppard JD (1993). Improved volume control for self-cycling fermentations. Can. J. Chem. Eng..

[35] Crosman JT, Pinchuk RJ, Cooper DG (2002). Enhanced biosurfactant production by *Corynebacterium alkanolyticum* ATCC 21511 using self-cycling fermentation. J. Am. Oil Chem. Soc..

[36] Levy S, Barkai N (2009). Coordination of gene expression with growth rate: A feedback or a feed-forward strategy?. FEBS Lett..

[37] Müller J, Dawson PS (1968). The operational flexibility of the phased culture technique, as observed by changes in the cell cycle of *Candida utilis*. Can. J. Microbiol..

[38] Mookerjee, S. Directing precursor flux to optimize cis,cis-muconic acid production in *Saccharomyces cerevisiae*. (Concordia University, 2016).

[39] Miller GL (1959). Use of dinitrosalicylic acid reagent for determination of reducing sugar. Anal. Chem..

[40] Bollmann, A., French, E. & Laanbroek, H. J. Isolation, cultivation, and characterization of ammonia-oxidizing bacteria and archaea adapted to low ammonium concentrations. in *Methods in Enzymology* vol. 486 55–88 (Academic Press Inc., 2011). 10.1016/B978-0-12-381294-0.00003-1 21185431

[41] Davison SA, den Haan R, van Zyl WH (2016). Heterologous expression of cellulase genes in natural *Saccharomyces cerevisiae* strains. Appl. Microbiol. Biotechnol..

[42] Cankorur-Cetinkaya A (2012). A novel strategy for selection and validation of reference genes in dynamic multidimensional experimental design in yeast. PLoS ONE.

[43] Teste MA, Duquenne M, François JM, Parrou JL (2009). Validation of reference genes for quantitative expression analysis by real-time RT-PCR in *Saccharomyces cerevisiae*. BMC Mol. Biol..

[44] Untergasser A (2012). Primer3-new capabilities and interfaces. Nucleic Acids Res..

[45] Livak KJ, Schmittgen TD (2001). Analysis of relative gene expression data using real-time quantitative PCR and the 2-ΔΔCT method. Methods.

