# Supplementary material for: The influence of self-cycling fermentation long- and short-cycle schemes on Saccharomyces cerevisiae and Escherichia coli
Source: Sci Rep. 2022 Aug 1;12:13154. doi: 10.1038/s41598-022-16831-x (PMC9343364; doi:10.1038/s41598-022-16831-x)
Supplement: Supplementary file 1 — Supplementary Figures. [file 41598_2022_16831_MOESM1_ESM.docx]

# Supplementary Information *for*

# The influence of self-cycling fermentation long- and short-cycle schemes on *Saccharomyces cerevisiae* and *Escherichia coli*

Yusheng Tan^1^, Lisa Y. Stein^2^, Dominic Sauvageau^1*^

^1^Department of Chemical and Materials Engineering, University of Alberta, Edmonton, Alberta, Canada

^2^Department of Biological Sciences, University of Alberta, Edmonton, Alberta, Canada

***Address correspondence to:**

Dominic Sauvageau, dominic.sauvageau@ualberta.ca

13-370, Department of Chemical and Materials Engineering, University of Alberta, 9211 - 116 St. NW, Edmonton, AB, Canada, T6G 1H9

### Additional Information

## Supplementary Information

Supplementary Information includes: Nitrogen Consumption in *S. cerevisiae* SCF Operation and Results and Discussion for *E. coli* BR Operation (text), replicate CER results of *S. cerevisiae* and *E. coli* grown in long- and short-cycle SCF operation (Supplementary Fig. S1), additional parameters for *S. cerevisiae* undergoing SCF long- and short-cycle schemes (Supplementary Fig. S2), relative expression of selected *S. cerevisiae* cyclin genes in biological replicate experiments (Supplementary Fig. S3), additional parameters for *E. coli* undergoing SCF long- and short-cycle schemes (Supplementary Fig. S4), growth of *E. coli* MG1655 in extended batch operation (Supplementary Fig. S5), **s**chematic of the batch reactor configuration used for this study (Supplementary Fig. S6), ammonium concentration in *S. cerevisiae* SCF long cycle 10 (Supplementary Fig. S7),.

### Nitrogen Consumption in S. cerevisiae SCF Operation

The nitrogen source, ammonium, was in excess throughout *S. cerevisiae* SCF long cycle 10 (Supplementary Fig. S7), and it was not the limiting nutrient during *S. cerevisiae* SCF operation.

### Results and Discussion for E. coli BR Operation

*E. coli* MG1655 was grown in a batch set-up for over 24 h. Two CER local minima were observed, one at 2 h and another at 7.5 h (Supplementary Fig. S5a). OD_600_ reached its maximum at 7.5 h, as CER reached a local minimum, and remained at approximately 2.5 for the remainder of the experiment (Supplementary Fig. S5b). Glucose was consumed rapidly until approximately 9 h, corresponding to the second CER maximum, after which point it remained relatively constant (Supplementary Fig. S5b). Glucose, which was expected to be the limiting nutrient, was not exhausted even after an extension of the operation well into stationary phase. Therefore, the abundance of nitrogen, in the form of ammonium, nitrate and nitrite, was examined; nitrogen compounds were found to remain abundant during the entire batch operation (Supplementary Fig. S5c). Additional calcium, iron, or yeast extract were added to fresh media in independent experiments; and these approaches did not lead to glucose exhaustion at the end of batch operation.

The two local minima in CER are consistent with observations in a prior *E. coli* study^1^, likely resulting from metabolic stalling or transitions between metabolic regimes. Potentially, the incomplete consumption of glucose at the end of batch operation was not attributed to a deprivation of a limiting nutrient. It was more likely due to an inhibitory effect of excessive intermediate metabolites (the bacterial Crabtree effect^2^): some organic acids might be produced in large amounts from rapid growth in the early stages of batch operation, potentially inhibiting the subsequent stages of growth and preventing the full consumption of glucose. The previous *E. coli* work observed complete glucose consumption once CER stopped decreasing, with similar initial nutrient conditions, however, with a different strain of *E. coli* (ATCC 11303)^1^.


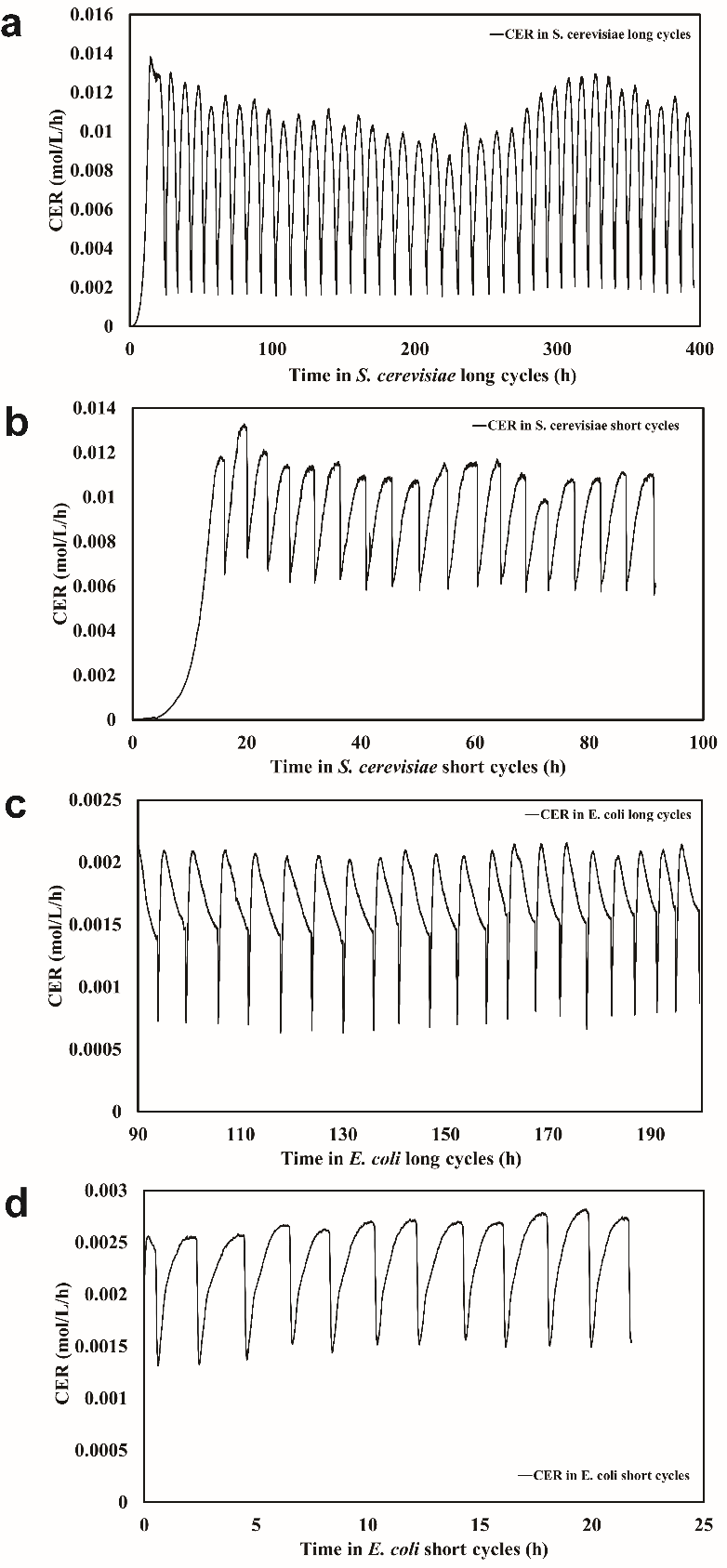


**Figure S1.** Replicate CER results of *S. cerevisiae* and *E. coli* grown in long- and short-cycle SCF operation. CER in (**a**) *S. cerevisiae* long-cycle operation, (**b**) *S. cerevisiae* short-cycle operation, (**c**) *E. coli* long-cycle operation, (**d**) *E. coli* short-cycle operation.


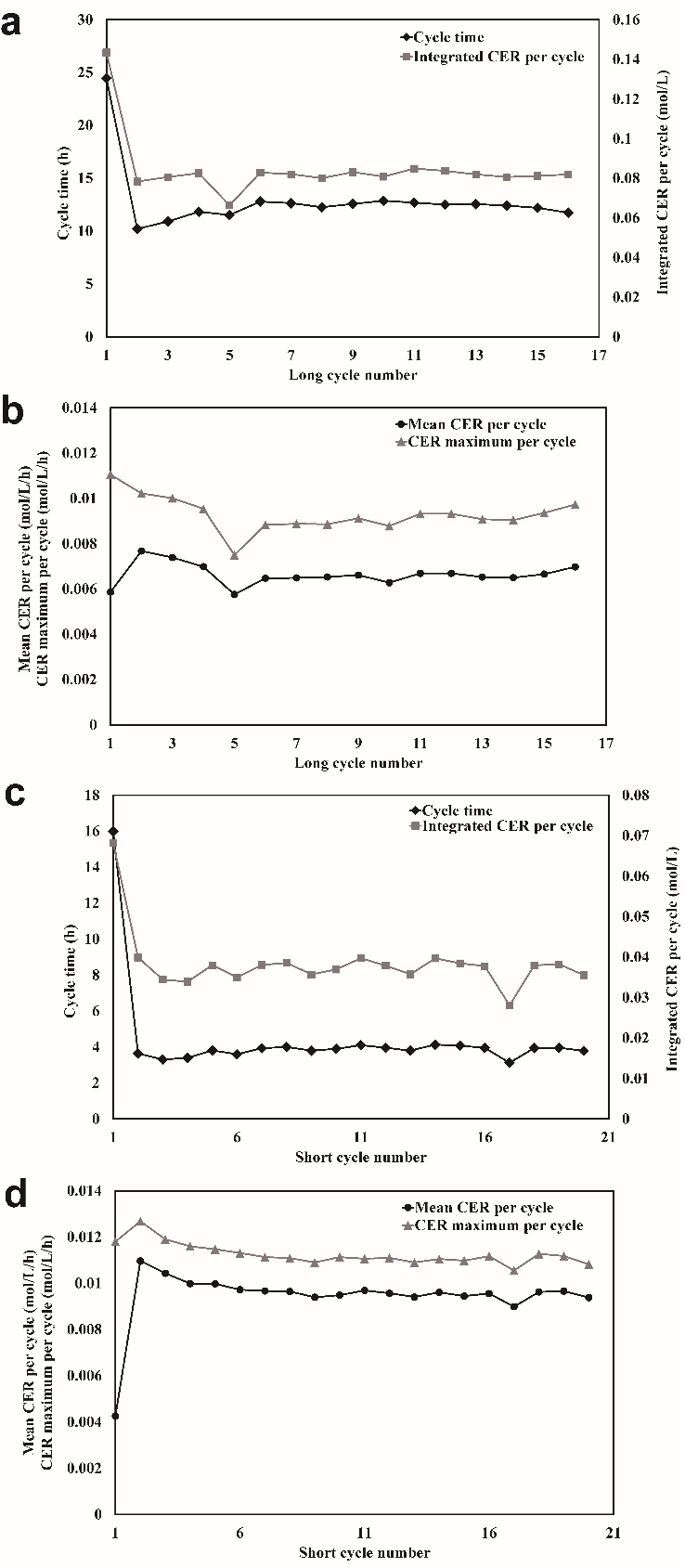


**Figure S2.** Additional parameters for *S. cerevisiae* undergoing SCF long- and short-cycle schemes. Long-cycle operation: (**a**) Long cycle time and integrated CER per long cycle, (**b**) mean CER per long cycle and CER maximum per long cycle. Short-cycle operation: (**c**) Short cycle time and integrated CER per short cycle, (**d**) mean CER per short cycle and CER maximum per short cycle.


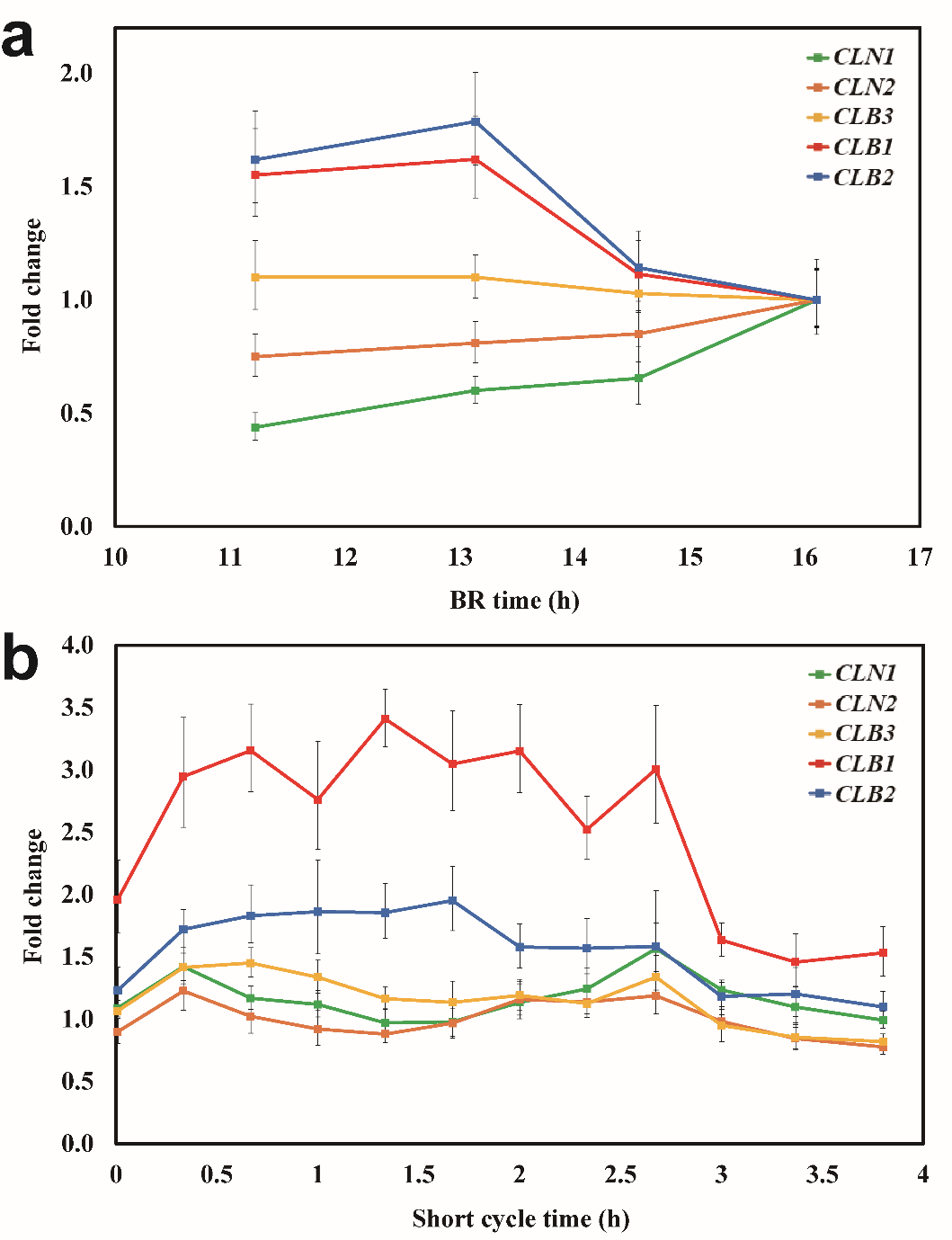


**Figure S3.** Relative expression of selected *S. cerevisiae* cyclin genes in biological replicate experiments. (**a**) Fold changes of *CLN1*, *CLN2*, *CLB3*, *CLB1,* and *CLB2* during BR late-log phase. (**b**) Fold changes of the same cyclin genes during SCF short cycle 21. *ACT1* and *ALG9* were used as reference genes, and a sample collected at 16.2 h during BR was used as the reference sample. Error bars show one standard deviations (n = 3).


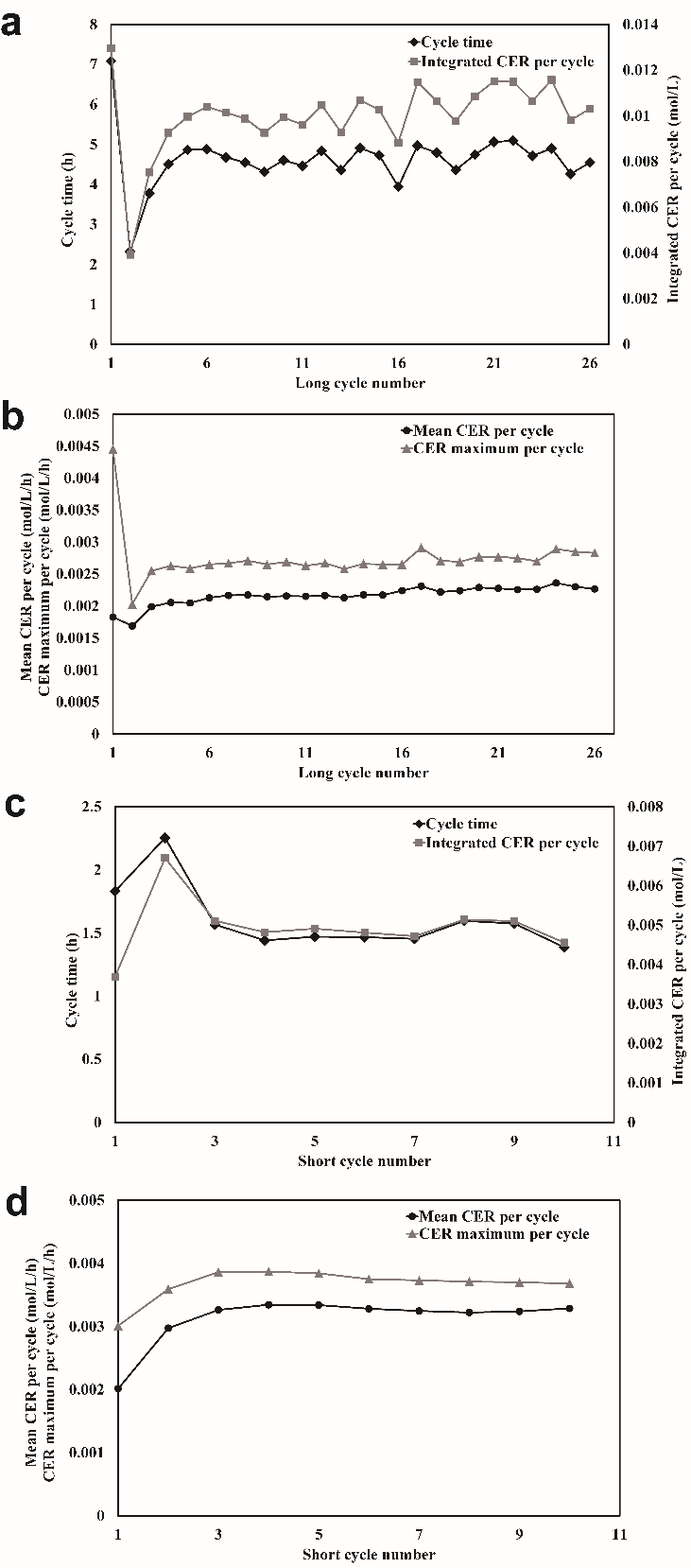


**Figure S4.** Additional parameters for *E. coli* undergoing SCF long- and short-cycle schemes. Long-cycle operation: (**a**) Long cycle time and integrated CER per long cycle, (**b**) mean CER per long cycle and CER maximum per long cycle. Short-cycle operation: (**c**) Short cycle time and integrated CER per short cycle, (**d**) mean CER per short cycle and CER maximum per short cycle.


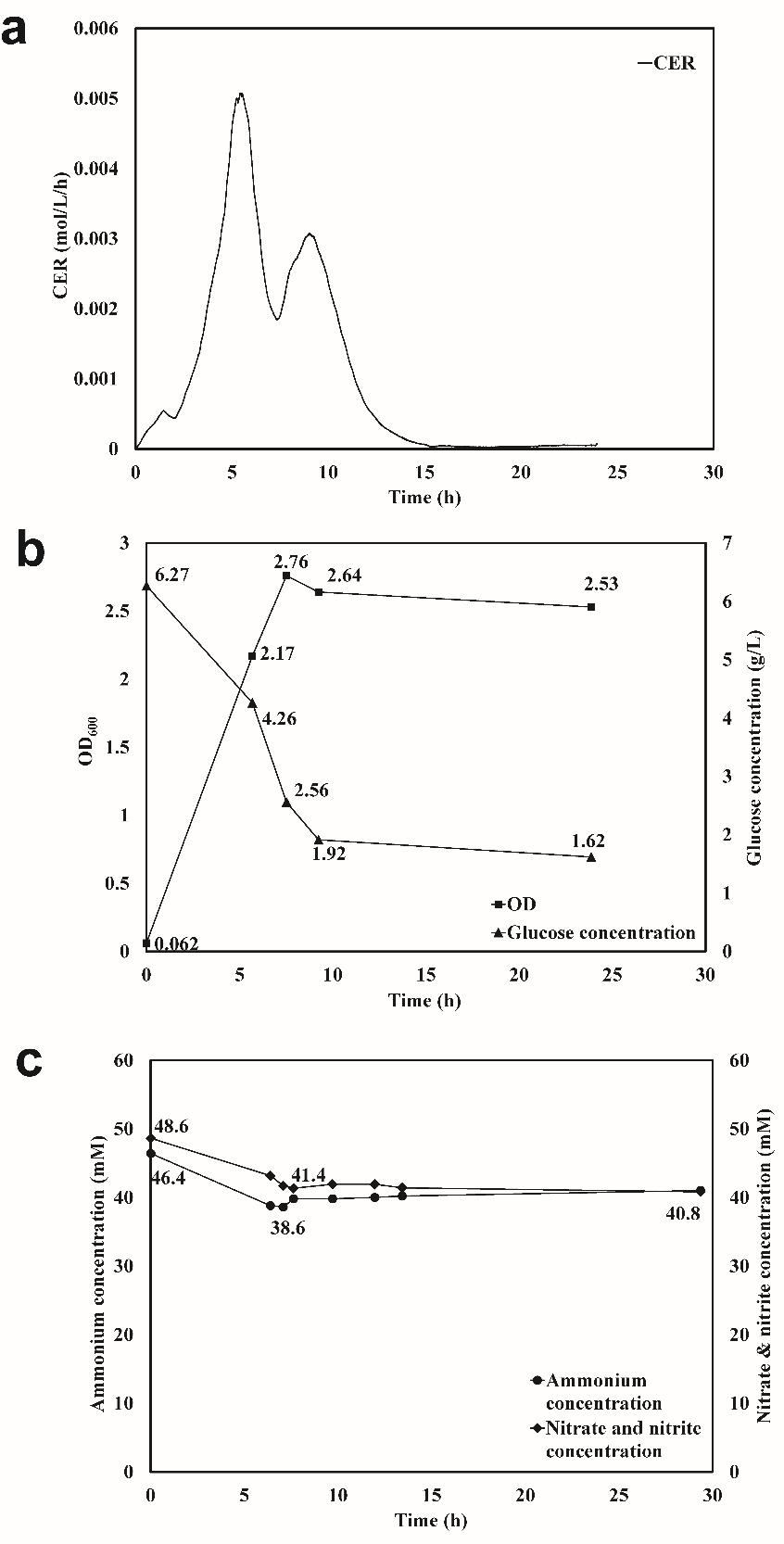


**Figure S5.** Growth of *E. coli* MG1655 in extended batch operation. (**a**) Carbon dioxide evolution rate (CER). (**b**) OD_600_ and glucose concentration. (**c**) Concentrations of ammonium, and combined nitrate and nitrite.


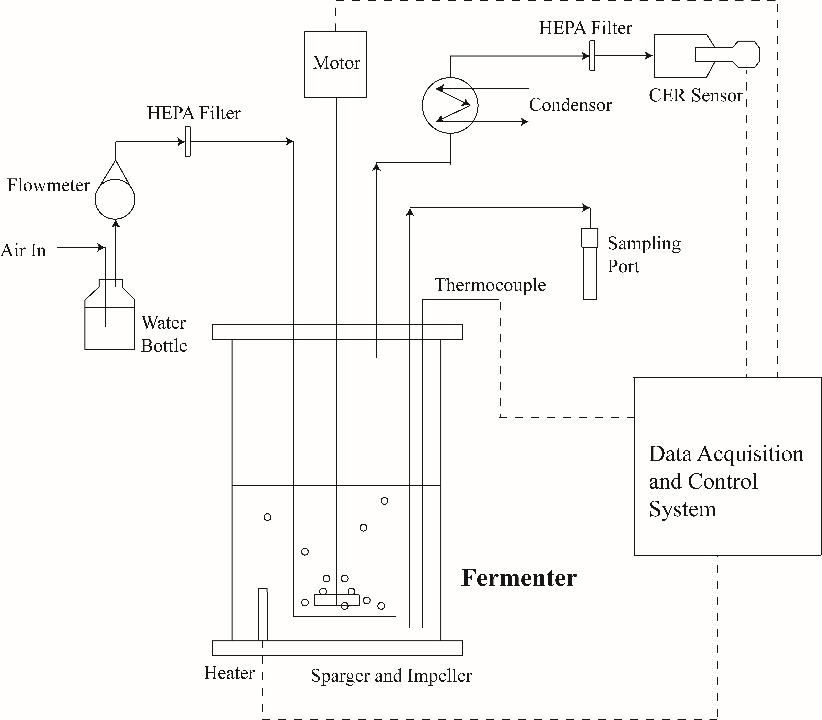


**Figure S6.** Schematic of the batch reactor configuration used for this study.


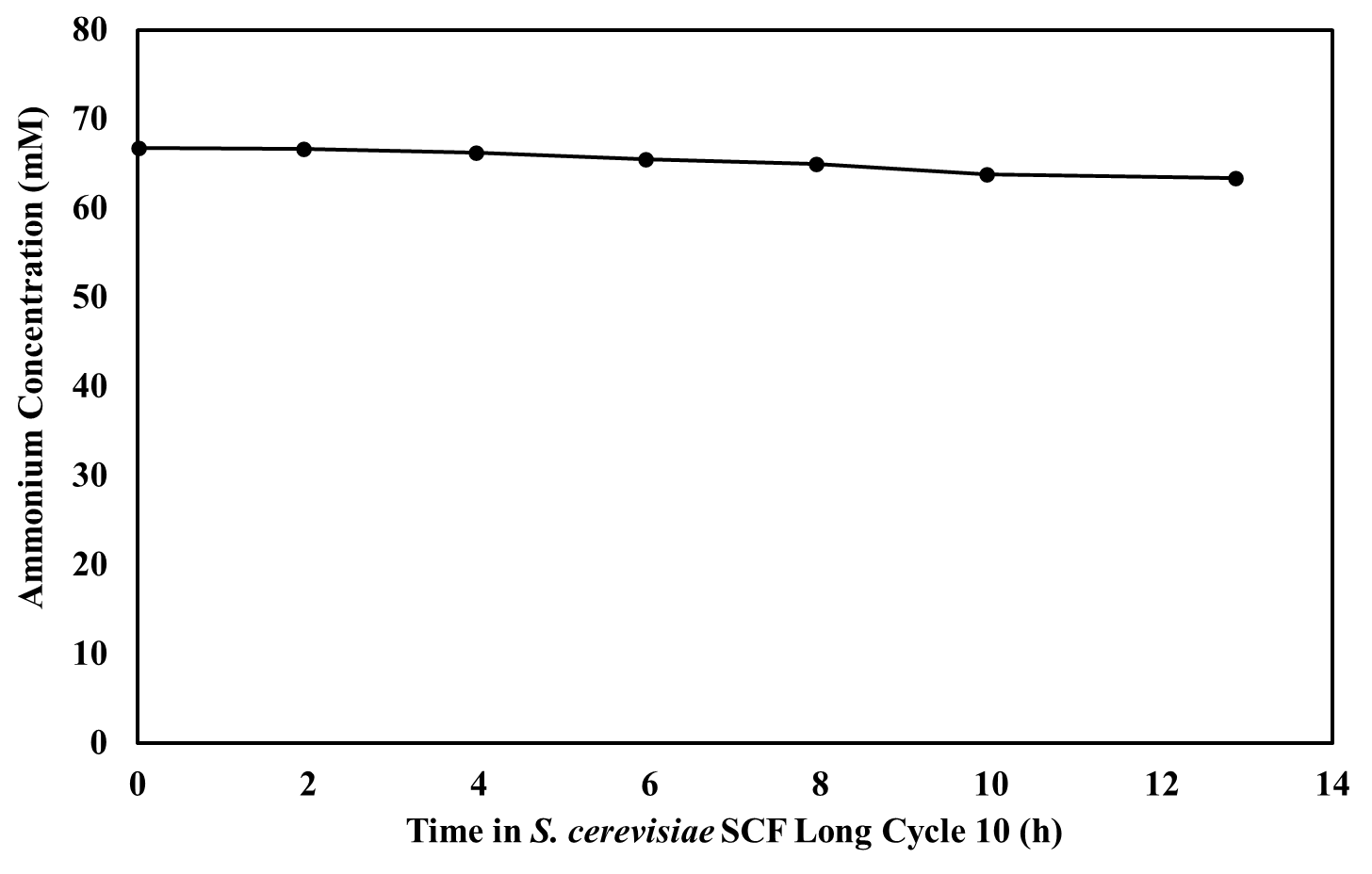


**Figure S7.** Ammonium concentration in *S. cerevisiae* SCF long cycle 10.

## References for Supplementary Information

1. Sauvageau, D., Storms, Z. & Cooper, D. G. Synchronized populations of *Escherichia coli* using simplified self-cycling fermentation. *J. Biotechnol.* **149**, 67–73 (2010).

2. Mustea, I. & Muresian, T. Crabtree effect in some bacterial cultures. *Cancer* **20**, 1499–1501 (1967).
